# Association of DNA methyltransferase polymorphisms with breast cancer: a nested case‒control study of the Arkansas Rural Community Health study

**DOI:** 10.1186/s12885-026-15695-y

**Published:** 2026-02-10

**Authors:** Sarah A. Mayberry, Ping-Ching Hsu, Hui-Yi Lin, Lora J. Rogers, Shelbie D. Stahr, L. Joseph Su

**Affiliations:** 1 Peter O’Donnell Jr. School of Public Health, UT Southwestern Medical Center, Dallas, TX USA; 2 Fay W. Boozman College of Public Health, University of Arkansas for Medical Sciences, Little Rock, AR USA; 3 Biostatistics and Data Science Program, School of Public Health, Louisiana State University Health Sciences Center, New Orleans, New Orleans, LA USA; 4 Department of Biochemistry, College of Medicine, University of Arkansas for Medical Sciences, Little Rock, AR USA

**Keywords:** DNA methyltransferase, Breast cancer, Single-nucleotide polymorphism, Haplotype

## Abstract

**Background:**

Breast cancer remains the most commonly diagnosed cancer and a leading cause of cancer-related mortality among women in the United States. While many risk factors have been identified, a substantial proportion of breast cancer cases occur in individuals without known risk profiles, underscoring the need to investigate novel genetic and epigenetic contributors. DNA methylation, an epigenetic modification regulated by DNA methyltransferase (DNMT) enzymes, plays a critical role in gene expression and genomic stability.

**Methods:**

This nested case‒control study, conducted within the Arkansas Rural Community Health study (ARCH) cohort, examined the associations between polymorphisms in *DNMT1*,* DNMT3A*, and *DNMT3B* and breast cancer risk. Using TaqMan genotyping and genome-wide association analysis in a sample of 2407 participants (967 cases and 1440 controls), we assessed both individual single-nucleotide polymorphisms (SNPs) and haplotypes.

**Results:**

*DNMT3A* SNP rs7605753 was significantly associated with increased breast cancer risk according to a recessive model (adjusted odds ratio [aOR]: 1.30; 95% confidence interval [CI]: 1.06, 1.59). Haplotype analysis revealed that *DNMT3A* haplotype TACGA was associated with increased breast cancer odds compared to the referent haplotype CGCGA (aOR: 1.42; 95% CI: 1.13, 1.79). Stratified analyses indicated that haplotypes CTACGA and CTGCAA were significantly associated with increased breast cancer risk among Black participants, with larger effect estimates than in White participants. No significant associations were observed for *DNMT1* or *DNMT3B* variants.

**Conclusions:**

These findings suggest that genetic polymorphisms in *DNMT3A* may contribute to breast cancer susceptibility and that racial differences in haplotype distribution and associated risk merit further investigation. Understanding these genetic associations could enhance personalized risk profiling and inform future breast cancer prevention strategies, particularly in understudied and diverse populations.

**Supplementary Information:**

The online version contains supplementary material available at 10.1186/s12885-026-15695-y.

## Background

To date, data suggest about one in every eight women in the United States will be diagnosed with breast cancer during their lifetime [1]. Breast cancer is the most diagnosed and second leading cause of cancer-related death among women in the United States. While mortality has declined due to early detection and treatment, incidence rates have shown a modest but steady increase over the past two decades [2]. Despite extensive research and the identification of numerous risk factors, about 50% of breast cancer cases occur in women who exhibit no apparent risk factors other than their age and sex, according to the World Health Organization [3]. Epigenetics, the study of heritable changes in gene expression that do not involve alterations to the underlying DNA sequence, is an area of growing interest in cancer epidemiology [4–6]. An important type of epigenetic modification is DNA methylation, the addition of a methyl group to a cytosine at the C5 position along the DNA sequence at a cytosine-phosphate-guanine (CpG) dinucleotide pair, transforming cytosine to 5-methylcytosine. These patterns are critical, as they control gene expression levels and differentiate cell types [7]. Aberrant DNA methylation patterns have been linked to cancer, including breast cancer, as they significantly alter gene expression, ranging from the silencing of tumor suppression genes to the overexpression of proliferation genes, and contribute to chromosomal instability [8]. 

DNA methylation is controlled by a class of epigenetic regulatory enzymes referred to as DNA methyltransferases (DNMTs). Five classes of DNMTs exist, three of which have direct enzymatic activity with DNA methylation: *DNMT1*,* DNMT3A*, and *DNMT3B* [9]. There are two principal activities in DNA methylation: establishment of DNA methylation patterns and maintenance of these patterns. Based on their activity, *DNMT3A* and *DNMT3B* have been classified as *de novo* methyltransferases, mainly controlling DNA methylation pattern establishment, and *DNMT1* as a maintenance methyltransferase [9]. However, numerous studies have demonstrated that *DNMT3A* and *DNMT3B* also exhibit maintenance methyltransferase activity, with mutations in these enzymes associated with tumor progression [10, 11]. Single-nucleotide polymorphisms (SNPs) are a common type of genetic mutation in which there is a change in a single-nucleotide along the DNA sequence. While some of these polymorphisms result in silent mutations, others can lead to significant phenotypic effects, affecting gene function, expression, or DNA stability [12]. Given the critical role of DNMTs in maintaining genomic stability and regulating gene expression, there is great interest in understanding how SNPs in these genes may influence cancer susceptibility, progression, and treatment. This knowledge is crucial for advancing precision medicine and addressing disparities in breast cancer outcomes for individuals with DNMT genetic mutations.

Genetic polymorphisms in DNA methyltransferases have been studied for their association with breast cancer risk in a limited number of populations, primarily among British and Han Chinese women [1, 13]. These studies identified various SNPs in *DNMT1*, *DNMT3A*, and *DNMT3B* genes that are associated with breast cancer risk development [1, 13]. However, a meta-analysis of these studies revealed differing results depending on the population studied and the specific SNPs analyzed [14]. These analyses have largely focused on single SNPs, potentially limiting the associations with breast cancer risk. In contrast, haplotype analysis explores clusters of nearby nucleotide variants that tend to be inherited together, creating larger genomic mutations that are likely to affect cancer susceptibility. To address this knowledge gap, we conducted a nested case‒control study within the Arkansas Rural Community Health study (ARCH) cohort to analyze these genetic polymorphisms and assess their consequent associations with breast cancer.

## Methods

### Data

We conducted a nested case‒control study consisting of 1484 breast cancer cases and 2154 controls from the Arkansas Rural Community Health study (ARCH), an ongoing prospective cohort comprised of about 26,000 women living throughout the state of Arkansas. ARCH enrolled participants between 2007 and 2013 to primarily study breast cancer and rural community health due to Arkansas’ unique landscape exhibiting high rurality and socioeconomic diversity. At the time of enrollment, participants completed a baseline questionnaire, underwent anthropologic measurements, and provided saliva samples. In 2018, genomic DNA was isolated from saliva specimens using DNA Genotek’s prepITL2P DNA Extraction Kit (Ontario, Canada) according to the manufacturer’s instructions. Genomic DNA was evaluated and quantified using a Nanodrop UV spectrometer (Thermo Fisher Scientific, Wilmington, DE).

Whole-genome amplification was conducted in the UAMS Genomics Core Facility using Qiagen REPLI-g Kits, which incorporate the method of multiple displacement amplification with input of 50 nanograms of genomic DNA per reaction. Amplified samples were purified and quantified on a Nanodrop 2000 (Wilmington, DE) before being plated for genotyping. The TaqMan method was used for SNP genotyping [15]. The polymorphisms were analyzed with the TaqMan SNP genotyping assays (Thermo Fisher Scientific, Waltham, MA, USA), with SNPs chosen based on a combination of prior research and preliminary data. Primers and probe mix were available as premade and validated TaqMan genotyping assays, and all PCR reactions were carried out with the TaqMan Genotyping Master Mix. Reactions were headed to 95 °C for 10 s and 60 °C for 1 min. PCR amplification was followed by allelic discrimination plate reading and analysis. For quality control, blinded repeats of approximately 5% of samples were included. SNPs with call rate ≥ 90% were retained, with *DNMT3A* SNP rs11892646 being the sole exception as its call rate (89.77%) was marginally lower than the threshold.

In our study, we excluded individuals with incomplete or ambiguous data on our variables of interest. We excluded individuals who had self-reported breast cancer diagnosis yet had no record or linkage to the Arkansas Cancer Registry to minimize misclassification. Additionally, we excluded individuals with incomplete data for any *DNMT1*, *DNMT3A*, or *DNMT3B* SNPs genotyped to maximize the integrity of the SNP sample data and allow for haplotype analysis. Finally, individuals with missing data on important covariates, including incomplete BMI measurements, missing racial designations, and inconclusive breastfeeding history, were excluded from the analysis, resulting in a final sample size of 2407 (66.16%), including 967 cases and 1440 controls.

### Analysis

Demographic characteristics of the study population were examined and compared between cases and controls using t-tests for continuous variables and chi-square tests for categorical variables. We examined variables collected at enrollment, including age as of 2016, self-reported race, educational attainment level, BMI, history of giving birth, and history of breastfeeding.

For each DNA methyltransferase, we conducted analyses on individual SNPs as well as a haplotype analysis. Each SNP was assessed for Hardy‒Weinburg equilibrium (HWE) using a Pearson chi-square (X^2^) statistic in the control population. Given the large population size and heterogeneity of population demographics, a p-value of 0.01 was used to assess deviations from HWE. Additionally, the minor allele frequency (MAF) was computed both in the entire population and by race to see the major-to-minor allele distribution. For inclusion in the SNP and haplotype analysis, the threshold for MAF was set at 5% or greater. Only those SNPs conforming to HWE and MAF criteria were included in the SNP and haplotype analysis. For individual SNP association analyses, conventional analyses only use the additive model based on the selected allele for each SNP. Previous studies showed that this additive-mode-only approach can cause false negativity, so multiple inheritance modes (additive, dominant, and recessive) should be taken into consideration [16, 17]. Thus, SNPs were analyzed using dominant, additive, and recessive models using the *SNPassoc* package in R Studio; inheritance patterns with the lowest p-value were reported [18]. Haplotype analysis was conducted using a generalized linear regression model for haplotypes, available in the *haplo.stats* package, with a haplotype detection frequency set at 0.02 [19]. Due to population heterogeneity, further analysis was conducted, stratifying by race to further examine associations, particularly for *DNMT3A* haplotypes. For analysis with population stratification, a haplotype detection of 0.04 was used and inclusion criteria for SNPs were determined based on MAF and HWE testing per racial category. Since individuals categorized as race ‘Other’ comprised only 2.45% of our sample population, only those categorized as ‘White’ and ‘Black’ were included in the haplotype analysis stratified by population. All analyses were conducted using both crude and fully adjusted logistic regression models, with fully adjusted models including estimated age as of 2016, history of breastfeeding, parity, education, race, and BMI. Our study was limited in analysis as information on BRCA gene mutations, a well-defined risk factor for breast cancer, was not available. In this study, exploratory analyses were conducted with a significance threshold set at *p* < 0.05, so no formal statistical adjustment for multiple comparisons was performed.

## Results

Our sample population, as of 2016, averaged 61.3 years of age, with cases being almost 2 years older on average than controls (*p* < 0.01, Table 1). A significant majority of the individuals in our population were White (84.42%), with 13.13% Black and 2.45% other races/ethnicities. BMI was slightly higher and had greater variation in controls compared to cases, resulting in a statistically significant difference (*p* = 0.02, Table 1). The educational attainment distribution did not significantly differ between cases and controls, with the majority having some college or a college degree. Regarding parity and breastfeeding status, 86.79% of the individuals had a history of giving birth, whereas only 45.24% had a history of breastfeeding (Table 1**)**. These distributions did not significantly differ between cases and controls.

**Table 1:** Sociodemographic and descriptive statistics of study participants

Subject Characteristics		Total (*N* = 2407)	Cases (*N* = 967)	Controls (*N* = 1440)	*P*-value
		Mean (STD)	Mean (STD)	Mean (STD)	
Estimated Age 2016 (years)		61.25 (11.62)	62.4 (11.73)	60.47 (11.49)	< 0.001
BMI (kg/m2)		28.80 (7.02)	28.59 (6.39)	28.94 (7.42)	0.022
		N (%)	N (%)	N (%)	
Race	White	2032 (84.4)	810 (83.8)	1222 (84.9)	0.021
	Black	316 (13.1)	123 (12.7)	193 (13.4)	
	Other	59 (2.5)	34 (3.5)	25 (1.7)	
Education	Less than high school	84 (3.5)	27 (2.8)	57 (3.9)	0.409
	High school graduate	481 (20.0)	192 (19.8)	289 (20.1)	
	Some college	817 (33.9)	339 (35.1)	478 (33.2)	
	College graduate	1025 (42.6)	409 (42.3)	616 (42.8)	
Ever given birth	Yes	2089 (86.8)	831 (85.9)	1258 (87.4)	0.342
	No	318 (13.2)	136 (14.1)	182 (12.6)	
Breast Fed	Yes	1089 (45.2)	435 (45.0)	654 (45.4)	0.867
	No	1318 (54.8)	532 (55.0)	786 (54.6)	

Three SNPs were analyzed in *DNMT1*, with all SNPs satisfying Hardy‒Weinberg equilibrium (HWE) under the inclusion criterion of *p* > 0.01 (Table 2). Six SNPs were examined in *DNMT3A*, of which rs2304429 was the only one that deviated from HWE (*p* < 0.001, Table 2). However, this HWE deviation was resolved when data were stratified by race, suggesting potential population stratification for this SNP (Table 2) [20]. Additionally, potential population stratification for rs2304429 was indicated by the difference in MAF by race, with an MAF of 41.8% in Whites compared to an MAF of 21.5% in Blacks (Table 2). As such, we performed a *DNMT3A* haplotype analysis excluding rs2304429 and then further conducted a population-stratified haplotype analysis with the inclusion of rs2304429.

**Table 2:** Hardy‒Weinberg equilibrium (HWE) and minor allele frequency (MAF) of DNMT SNPs

Gene	rs Number	Alleles (Major/Minor)	MAF %	MAF % White/Black	HWE Controls (*p*-value)	HWE Controls White/Black (*p*-value)
*DNMT1*	rs8101626	A/G	40.9%	44.8%/23.1%	0.022	0.562/0.011
	rs2290684	A/G	49.7%	49.6%/49.2%	0.171	0.530/0.084
	rs11880388	G/A	49.4%	49.4%/50.0%	0.140	0.493/0.061
*DNMT3A*	rs2304429	T/C	46.5%	41.8%/21.5%	< 0.001	0.290/0.834
	rs12991495	T/C	29.0%	30.5%/13.3%	0.442	1.000/1.000
	rs7605753	G/A	44.1%	46.9%/35.0%	0.669	0.773/0.206
	rs11892646	C/T	14.1%	12.1%/28.2%	0.064	0.064/0.400
	rs7575625	A/G	44.7%	43.7%/45.3%	0.201	0.224/0.886
	rs10196635	A/T	8.7%	9.4%/7.8%	1.000	1.000/1.000
*DNMT3B*	rs2424905	C/T	48.8%	44.8%/15.0%	< 0.001	0.018/0.747
	rs2424910	G/T	1.6%	0.3%/8.7%	0.295	1.000/1.000
	rs17123590	G	0.0%	0.0%/0.0%	----	-----
	rs6058896	C/T	6.1%	5.5%/9.8%	-------	--------

Four SNPs were analyzed in *DNMT3B*. HWE could not be calculated for rs17123590 due to the absence of a minor allele nor for rs6058896 due to the lack of a homozygous recessive genotype (TT) existing in the study population. Rs2424905 significantly deviated from HWE, with *p* < 0.001 (Table 2). However, in stratifying by race, this deviation was resolved, suggesting potential population stratification for this SNP (Table 2). Additionally, rs2424905 MAF was higher in Whites (44.8%) compared to Blacks (15.0%), further supporting potential population stratification. For rs2424910, the MAF fell below the threshold for inclusion in the SNP and haplotype analysis (1.6%, Table 2). However, when the MAF was examined by race, Blacks had a higher MAF frequency which fell within the threshold criteria (8.7%, Table 2). With these numerous deviations from HWE and MAF for *DNMT3B* SNPs, only rs2424905 was included for SNP analysis, and no haplotype analysis for *DNMT3B* was completed.

Both dominant, additive, and recessive model types were evaluated, with the most likely model of inheritance listed in Table 3. Using the chosen models, each SNP was analyzed for association with breast cancer odds in crude and fully adjusted models.

**Table 3:** Associations of DNMT SNPs with breast cancer

Gene	rs Number	Alleles (Major/Minor)	Model	Crude OR [95% CI]	Adjusted OR [95% CI]
*DNMT1*	rs8101626	A/G	Dominant	1.04 [0.87, 1.23]	1.05 [0.88, 1.25]
	rs2290684	A/G	Additive	1.02 [0.91, 1.14]	1.01 [0.90, 1.13]
	rs11880388	G/A	Dominant	1.04 [0.86, 1.25]	1.03 [0.85, 1.24]
*DNMT3A*	rs2304429	T/C	Recessive	1.02 [0.85, 1.24]	1.06 [0.86, 1.30]
	rs12991495	T/C	Dominant	0.89 [0.76, 1.05]	0.87 [0.74, 1.03]
	rs7605753	G/A	Recessive	1.27 [1.04, 1.55]	1.30 [1.06, 1.59]
	rs11892646	C/T	Dominant	1.06 [0.88, 1.28]	1.07 [0.89, 1.30]
	rs7575625	A/G	Recessive	0.87 [0.71, 1.07]	0.87 [0.70, 1.07]
	rs10196635	A/T	Additive	1.17 [0.96, 1.43]	1.17 [0.96, 1.43]
*DNMT3B*	rs2424905	C/T	Dominant	1.15 [0.96, 1.38]	1.17 [0.96, 1.43]

No *DNMT1* SNPs or *DNMT3B* SNPs were significantly associated with breast cancer. *DNMT3A* SNP rs7605753 was significantly associated with increased breast cancer odds in a recessive model (AA vs. AG/GG), with an adjusted odds ratio of 1.30 (95% CI: 1.06, 1.59) (Table 3). All other *DNMT3A* SNPs were not significantly associated with breast cancer.

Three haplotypes were identified for *DNMT1* (Table 4); however, none of these haplotypes were significantly associated with breast cancer. For *DNMT3A*, CGCGA was chosen as the referent haplotype as a result of three considerations: relatively high expected frequency, consideration of each SNP’s major allele, and the SNP’s direction of association with breast cancer odds. Since the minor alleles of rs12991495 and rs7575625 were associated with decreased breast cancer odds, although not significantly (Table 3), they were considered appropriate for inclusion in the referent haplotype. Two haplotypes were significantly associated with increased breast cancer odds in adjusted models: TACAT and TACGA (Table 4). Compared to the referent haplotype, the TACAT haplotype, observed in 5.68% of the study population, was associated with a 40% increase in breast cancer odds (aOR: 1.40; 95% CI: 1.02, 1.92) (Table 4). The TACGA haplotype, present in 19.48% of the population, was associated with a 42% increase in breast cancer odds compared to the referent haplotype (aOR: 1.42; 95% CI: 1.13, 1.79) (Table 4). While other haplotypes had an increased association with breast cancer odds, none were statistically significant.

**Table 4:** Associations of *DNMT1* and *DNMT3A* haplotypes with breast cancer

Gene	Haplotype	Estimated Population %	Crude OR [95% CI]	Adjusted OR [95% CI]
*DNMT1*	rs8101626 – rs2290684 – rs11880388			
	AGA	49.5	1.0 [REF]	1.0 [REF]
	GAG	40.7	0.99 [0.88, 1.12]	1.00 [0.89, 1.13]
	AAG	9.38	0.92 [0.76, 1.12]	0.94 [0.76, 1.16]
*DNMT3A*	rs12991495 – rs7605753 – rs11892646 – rs7575625 – rs10196635			
	CGCGA	13.52	1.0 [REF]	1.0 [REF]
	CACAA	4.14	1.25 [0.85, 1.85]	1.25 [0.84, 1.86]
	CACAT	2.11	1.49 [0.88, 2.53]	1.51 [0.89, 2.57]
	CGCAA	4.92	1.36 [0.91, 2.05]	1.38 [0.91, 2.08]
	TACAA	12.42	1.23 [0.96, 1.57]	1.27 [0.99, 1.63]
	TACAT	5.68	1.40 [1.02, 1.92]	1.40 [1.02, 1.92]
	TACGA	19.48	1.39 [1.11, 1.75]	1.42 [1.13, 1.79]
	TGCAA	13.68	1.28 [0.93, 1.75]	1.29 [0.99, 1.63]
	TGCGA	7.53	1.13 [0.82, 1.55]	1.13 [0.83, 1.53]
	TGTAA	9.96	1.24 [0.96, 1.60]	1.27 [0.98, 1.66]

Haplotypes were also analyzed using population stratification methods (Table 5). Upon stratification by race, the haplotypes CTACGA, TTACAA, and TTACAT were significantly associated with increased breast cancer odds compared to the referent haplotype, TCGCGA, in Whites (Table 5). A similar rationale for choosing the referent haplotype was applied as in the haplotype analysis conducted without population stratification. Utilizing the same referent haplotype in Blacks, CTACGA and CTGTGA were found to be significantly associated with increased odds of breast cancer. Specifically, compared to the TCGCGA haplotype, the CTACGA haplotype was associated with a 5.72-fold increase in breast cancer odds after adjusting for covariates (95% CI: 1.34, 24.39) (Table 5). The referent haplotype, TCGCGA, was relatively infrequent in Blacks at 4.87% compared to 13.60% in Whites. To conduct more stable analysis, the most common haplotype in Blacks of this study population, CTGTAA, was then utilized to examine the association of haplotypes and breast cancer in this stratum (Table S1). Utilizing CTGTAA as the referent haplotype, CTACGA and CTGCAA remained significantly associated with increased breast cancer odds, with narrower confidence intervals estimating the measure of association. No other haplotypes were significantly associated with breast cancer in this stratum when CTGTAA was used as the referent haplotype.

**Table 5:** Associations of *DNMT3A* haplotypes with breast cancer utilizing population stratification

Race	Haplotype	Estimated Population %	Crude OR [95% CI]	Adjusted OR [95% CI]
rs2304429 - rs12991495 - rs7605753 - rs11892646 - rs7575625 -rs10196635				
White	TCGCGA	13.60	1.0 [REF]	1.0 [REF]
	CTACGA	15.20	1.43 [1.10, 1.85]	1.47 [1.13, 1.90]
	CTGCAA	8.48	1.19 [0.87, 1.63]	1.18 [0.86, 1.62]
	CTGTAA	5.00	1.33 [0.91, 1.94]	1.35 [0.92, 1.98]
	TTACAA	7.09	1.43 [1.03, 1.99]	1.48 [1.06, 2.07]
	TTACAT	5.87	1.45 [1.04, 2.03]	1.45 [1.03, 2.03]
Black	TCGCGA	4.87	1.0 [REF]	1.0 [REF]
	CTACGA	17.81	6.60 [1.52, 28.70]	5.72 [1.34, 24.39]
	CTGCAA	10.26	9.84 [2.16, 44.92]	8.62 [1.93, 38.45]
	CTGTAA	20.22	3.47 [0.83, 14.60]	2.98 0.73, 12.16]
	CTGTGA	4.51	6.73 [1.26, 36.14]	5.99 [1.37, 26.32]

## Discussion

### SNP analysis

No *DNMT1* or *DNMT3B* SNPs analyzed in our study were significantly associated with breast cancer. However, our findings of *DNMT1* genetic polymorphisms associated with breast cancer may be limited by the SNPs analyzed, as previous studies exploring *DNMT1* genetic polymorphisms found significant associations with breast cancer. Analysis of a central European Caucasian population by Kullmann et al. identified a significant reduction in breast cancer risk associated with the G allele of *DNMT1* SNP rs2228612, a SNP not analyzed in our study [13]. 

Ye et al. conducted a two-stage case‒control study examining *DNMT1* and *DNMT3B* genetic polymorphisms with breast cancer in Chinese women [21]. The study analyzed 1 SNP on *DNMT3B* that was included in our study, rs6058896, similarly finding no significant association with breast cancer risk. However, our study differed in that our population lacked homozygous recessive genotypes for rs6058896 [21]. 

The only SNP significantly associated with increased breast cancer odds in our study was rs7605753 on *DNMT3A*, with the AA genotype conferring a 30% increase in breast cancer odds compared to the GG or AG genotype (aOR: 1.30, 95% CI: 1.06, 1.59). Rs7605753 is an intron variant for *DNMT3A*, located at chromosome 2, position 25,270,318 (GRCh38.p14) [22]. This SNP has been associated with expression quantitative trait locus (eQTL) activity in two studies conducted by the Boyle Lab, [23, 24] suggesting a regulatory effect in gene expression. To our knowledge, no published studies on clinical significance have been conducted on this SNP, indicating a novel finding [22]. However, as this study did not adjust the significance level for multiple comparisons, these results should be interpreted as exploratory and hypothesis-generating, underscoring the need for further research into this SNP to understand its relation to cancer development and susceptibility.

Few studies have analyzed the association between *DNMT3A* genetic polymorphisms and breast cancer. Deroo et al. analyzed *DNMT3A* genetic polymorphisms with breast cancer using a case-cohort study design with participants in the Sister Study, analyzing 1 SNP that we also analyzed [25]. While the SNP was not significantly associated with breast cancer in either study, our studies differed in the direction of the effect. Deroo et al. found rs7575625 to have a hazard ratio of 1.05 (95% CI: 0.85, 1.29),^25^ whereas our study reported a nonsignificant inverse association with breast cancer odds (aOR: 0.87, 95% CI: 0.70, 1.07).

### Haplotype analysis without population stratification

In the haplotype analysis without population stratification, haplotypes TACGA and TACAT were significantly associated with increased breast cancer odds of 42% and 40%, respectively, compared to haplotype CGCGA.

Compared with CGCGA, TACGA had 2 different alleles: the major allele of rs12991495 and the minor allele of rs7605753. In the SNP analysis, the minor allele of rs7605753 had a statistically significant 30% increase in breast cancer odds (aOR: 1.30, 95% CI: 1.06, 1.59). For rs12991495 SNP analysis, the minor allele displayed a protective association compared to the major allele but this association with breast cancer odds did not reach statistical significance (Table 3). While not statistically significant in the SNP analysis, haplotype analysis revealed that this SNP may contribute to increased odds of breast cancer when combined with other polymorphisms, as TACGA had an increased odds of breast cancer compared to the analysis with SNP rs7605753 alone.

The other haplotype identified, TACAT, had 4 differing alleles from CGCGA: the same 2 alleles as TACAT, the major allele of rs7575625, and the minor allele of rs10196635. In the SNP analysis, the minor allele of rs7575625 had a nonsignificant protective effect on breast cancer odds. Rs10196635, while not statistically associated with breast cancer odds, was particularly interesting, as the estimated increase in breast cancer odds was 17% for every minor allele present (aOR: 1.17, 95% CI: 0.96, 1.43). The minor allele was very infrequent in the study population at only 6.15% (Table 2), potentially underpowering the study to detect any true associations present. These results spark interest in future research of this SNP, particularly for individuals with a homozygous recessive genotype. Additionally, this haplotype was interesting in that only 5.68% of the population was estimated to have this haplotype, producing wider variation in the 95% confidence interval estimation, yet still conferring statistically significant results. Therefore, this combination of SNPs warrants further investigation in populations with higher percentages of these SNPs present.

### Haplotype analysis with population stratification

Significant differences between haplotypes and breast cancer associations were observed by race upon inclusion of the *DNMT3A* SNP rs2304429, which indicated population stratification. As Whites constituted the majority of the study population, similar results occurred as those in the analysis without population stratification. For the White stratum, including SNP rs2304429, we found haplotype CTACGA associated with a 47% increase in breast cancer odds compared to TCGCGA (aOR: 1.47, 95% CI: 1.13, 1.90). According to the SNP analysis, the minor allele of rs2304429 was insignificantly associated with increased breast cancer odds (Table 3**)**, potentially explaining the 5% increase in breast cancer odds from TACGA to CTACGA.

In the Black stratum, CTACGA was significantly associated with increased odds of breast cancer; however, estimates had considerably less precision compared to the White stratum, likely due to the low frequency of the referent haplotype in the Black stratum at 4.87% compared to 13.60% in the White stratum. In this subgroup analysis, CTACGA was associated with a 5.72-fold increase in breast cancer odds compared to TCGCGA after adjusting for covariates (95% CI: 1.34, 24.39). Interestingly, CTACGA was more common in the Black stratum, with 17.73% of the population estimated to have the haplotype, compared to 15.21% in the White stratum. Another haplotype of interest was identified in this stratum: CTGCAA. This haplotype was associated with an 8.62-fold increase in breast cancer odds compared to TCGCGA (95% CI: 1.93, 38.45), an association not present in the White stratum.

CTGCAA has the major allele of rs7605753, an unusual result as the minor allele rs7605753 was the only SNP significantly associated with breast cancer in the SNP analysis (Table 3). However, examining the SNP by race, rs7605753 was not significantly associated with breast cancer in the Black stratum (aOR: 1.34, 95% CI: 0.63, 2.87) (Table S2). This result could indicate that there truly is no association between rs7605753 and breast cancer in the Black population of our study, or we failed to detect the association due to a small sample size of Black participants (*n* = 316) compared to White participants (*n* = 2032). This potential limitation highlights the importance for future studies to prioritize diversity and sufficient sampling so these exploratory findings in SNP and haplotypes associations by population may be sufficiently determined.

We also conducted *DNMT3A* haplotype analysis in the Black stratum using the most common haplotype identified in this population, CTGTAA, to increase stability and narrow confidence intervals for our analysis (Table S1). Utilizing this referent haplotype, CTACGA and CTGCAA remained significantly associated with increased breast cancer odds, with greater certainty of the association. CTACGA had an estimated 1.92-fold increase in breast cancer odds compared to CTGTAA after adjusting for covariates (95% CI:1.01, 3.63), indicating this haplotype is significantly associated with breast cancer regardless of racial background. CTGCAA had an estimated 2.89-fold increase in breast cancer odds compared to CTGTAA after adjusting for covariates (95% CI: 1.36, 6.12). This haplotype differed from the referent haplotype only by 1 allele: the major allele of rs11892646. In the SNP analysis, the major allele of rs11892646 had a nonsignificant increased association with breast cancer odds (aOR: 1.07, 95% CI: 0.89, 1.30). The haplotype analysis suggests rs11892646 may confer increased breast cancer odds that were masked in the overall analysis due to lack of population diversity, warranting further investigation into this SNP.

Our results suggest that in the general population, *DNMT3A* haplotype TACGA could be associated with increased breast cancer odds, with further large-scale studies needed to explore these exploratory results due to increased risk of false positive findings. Results also revealed important racial/ethnic differences upon population stratification, highlighting how aggregate results may conceal important association differences, as some populations have differing allele frequencies. This study was a majority White population, limiting statistical assessment of haplotypes among races, particularly for Black study participants. However, haplotype analysis with population stratification suggests that *DNMT3A* haplotype CTACGA could be significantly associated with increased breast cancer odds compared to TCGCGA, as Black women had an increased association (aOR: 5.72, 95% CI: 1.34, 24.39) compared to White women (aOR: 1.47, 95% CI: 1.13, 1.90).

While greater research is needed to assess these differences in measures of association according to race, a leading hypothesis may be potential gene‒environment interactions or epigenetic changes in this population due to factors related to systemic racism, such as environmental exposures, housing, or psychosocial stressors which may disparately affect Black women in our population. A study conducted by Jasmine M Miller-Kleinhenz et al. identified 5 differentially methylated CpG sites that were associated with breast carcinogenesis and contemporary redlining in breast cancer patients from Emory University Hospitals in Atlanta, Georgia. Their study revealed significant associations with this systemic racism practice and epigenetic age acceleration, providing evidence that social determinants and environmental exposures may contribute to one’s epigenome, affecting DNA methylation patterns and contributing to breast cancer development [26]. Additionally, a cross-sectional study in Baltimore found epigenetic differences in breast tumors attributable to neighborhood deprivation level, which was higher for Black women compared to their White counterparts [27]. These studies provide evidence that epigenetic modifications related to social and environmental influences negatively affect breast cancer, highlighting the need to research how these DNMT genetic polymorphisms may contribute to differing breast cancer risks in conjunction with these exposures.

## Conclusion

This study provides novel evidence that genetic variation in the *DNMT3A* gene, particularly the rs7605753 SNP and associated haplotypes, may contribute to breast cancer susceptibility in a diverse U.S. population. The identification of significant associations, including race-specific haplotype effects, highlights the importance of considering genetic ancestry in epigenetic research. These exploratory findings suggest that *DNMT3A* polymorphisms could play a meaningful role in breast cancer risk and support further investigations into gene‒environment interactions and the functional relevance of these variants. Understanding the contributions of epigenetic regulators such as *DNMT3A* may enhance risk prediction and inform targeted prevention strategies, especially in underserved and racially diverse communities.

## Supplementary Information

Additional File 1: Table S1. Associations of *DNMT3A* haplotypes with breast cancer among Black stratum utilizing CTGTAA as the referent haplotype.

Additional File 2: Table S2. Associations of *DNMT3A* SNPs with breast cancer utilizing population stratification.

## Data Availability

The data that support the findings of this study can be made available in a limited dataset format upon request to the corresponding author.

## Abbreviations

aOR: Adjusted odds ratio
ARCH: Arkansas Rural Community Health study
CI: Confidence Interval
CpG: Cytosine-phosphate-guanine
DNMT: DNA methyltransferase
HWE: Hardy-Weinburg equilibrium
MAF: Minor allele frequency
SNP: Single-nucleotide polymorphism
