# Supplementary material for: Association of DNA methyltransferase polymorphisms with breast cancer: a nested case‒control study of the Arkansas Rural Community Health study
Source: BMC Cancer. 2026 Feb 10;26:357. doi: 10.1186/s12885-026-15695-y (PMC12997830; doi:10.1186/s12885-026-15695-y)
Supplement: Supplementary file 1 — Additional File 1: Table S1. Associations of DNMT3A haplotypes with breast cancer among Black stratum utilizing CTGTAA as the referent haplotype. [file 12885_2026_15695_MOESM1_ESM.docx]

**Table S1.** Associations of *DNMT3A* haplotypes with breast cancer among Black stratum utilizing CTGTAA as the referent haplotype

| **Haplotype** | **Estimated Population %** | **Crude OR [95% CI]** | **Adjusted OR [95% CI]** |
| --- | --- | --- | --- |
| rs2304429 - rs12991495 - rs7605753 - rs11892646 - rs7575625 -rs10196635 | | | |
| CTGTAA | 20.40 | 1.0 [REF] | 1.0 [REF] |
| CTACAA | 9.36 | 0.87 [0.37, 2.05] | 1.00 [0.42, 2.35] |
| CTACGA | 17.75 | 1.90 [1.02, 3.54] | 1.92 [1.01, 3.63] |
| CTGCAA | 10.24 | 2.83 [1.37, 5.85] | 2.89 [1.36, 6.12] |
| CTGCGA | 8.69 | 1.00 [0.46, 2.18] | 0.93 [0.48, 2.40] |
| CTGTGA | 4.51 | 1.94 [0.66, 5.66] | 2.01 [0.68, 5.97] |
| TCGCGA | 4.86 | 0.29 [0.07, 1.21] | 0.34 [0.08, 1.37] |
