# Supplementary material for: Association of DNA methyltransferase polymorphisms with breast cancer: a nested case‒control study of the Arkansas Rural Community Health study
Source: BMC Cancer. 2026 Feb 10;26:357. doi: 10.1186/s12885-026-15695-y (PMC12997830; doi:10.1186/s12885-026-15695-y)
Supplement: Supplementary file 2 — Additional File 2: Table S2. Associations of DNMT3A SNPs with breast cancer utilizing population stratification. [file 12885_2026_15695_MOESM2_ESM.docx]

**Table S2.** Associations of *DNMT3A* SNPs with breast cancer utilizing population stratification

| **Race** | **rs Number** | **Alleles**  **(Major/Minor)** | **Model** | **Crude OR [95% CI]** | **Adjusted OR [95% CI]** |
| --- | --- | --- | --- | --- | --- |
| White | rs2304429 | T/C | Recessive | 1.04 [0.83, 1.31] | 1.06 [0.84, 1.33] |
|  | rs12991495 | T/C | Dominant | 0.89 [0.75, 1.07] | 0.88 [0.74, 1.05] |
|  | rs7605753 | G/A | Recessive | 1.27 [1.03, 1.57] | 1.30 [1.05, 1.61] |
|  | rs11892646 | C/T | Dominant | 1.16 [0.94, 1.44] | 1.16 [0.94, 1.44] |
|  | rs7575625 | A/G | Recessive | 0.88 [0.70, 1.10] | 0.87 [0.70, 1.10] |
|  | rs10196635 | A/T | Additive | 1.08 [0.88, 1.34] | 1.08 [0.87, 1.34] |
| Black | rs2304429 | T/C | Dominant | 0.91 [0.57, 1.45] | 0.89 [0.55, 1.44] |
|  | rs12991495 | T/C | Dominant | 0.75 [0.44, 1.28] | 0.86 [0.49, 1.50] |
|  | rs7605753 | G/A | Recessive | 1.18 [0.57, 2.44] | 1.34 [0.63, 2.87] |
|  | rs11892646 | C/T | Dominant | 0.74 [0.47, 1.17] | 0.72 [0.45, 1.16] |
|  | rs7575625 | A/G | Recessive | 0.76 [0.43, 1.37] | 0.71 [0.39, 1.30] |
|  | rs10196635 | A/T | Additive | 1.56 [0.87, 2.80] | 1.52 [0.83, 2.78] |
